# How Does Obesity Influence the Risk of Vertebral Fracture? Findings From the UK Biobank Participants

**DOI:** 10.1002/jbm4.10358

**Published:** 2020-03-26

**Authors:** Jin Luo, Raymond YW Lee

**Affiliations:** ^1^ School of Applied Sciences London South Bank University London UK; ^2^ Faculty of Technology University of Portsmouth Portsmouth UK

**Keywords:** BODY MASS INDEX, FAT MASS, FRACTURE RISK ASSESSMENT, SPINE, WAIST CIRCUMFERENCE

## Abstract

Obesity and osteoporotic‐related fractures are two common public health problems, although it is unclear how obesity affects the risk of vertebral fractures. The purpose of this study was to examine the association between different measures of obesity and the risk of vertebral fracture, and to establish the various clinical factors that can predict such risk. We analyzed data obtained from 502,543 participants in the UK Biobank (229,138 men and 273,405 women), aged 40 to 69 years. Imaging information was available in a subset of this cohort (5189 participants: 2473 men and 2716 women). We further examined how BMD and geometry of the vertebrae were related to body fat measures. It was shown that a larger waist circumference (WC), but not BMI, was associated with an increase in fracture risk in men, but in women, neither BMI nor WC affected the risk. Trunk fat mass, visceral adipose tissue (VAT) mass, and limb fat mass were negatively associated with vertebral body BMD and geometry in men and women. BMD and geometry are related to vertebral strength, but may not be directly related to the risk of fractures, which is also influenced by other factors. The binary logistic regression equation established in this study may be useful to clinicians for the prediction of vertebral fracture risks, and may provide further information to supplement the fracture risk assessment tool, which assesses general fracture risks. © 2020 The Authors. *JBMR Plus* published by Wiley Periodicals, Inc. on behalf of American Society for Bone and Mineral Research.

## Introduction

Obesity and osteoporosis are two very common public health problems. Obesity is sometimes thought to have a protective effect against osteoporotic fractures.^1^ A higher body weight may impose larger mechanical loading on bone and consequently help improve bone health and reduce the risk of fracture.^2^ However, recent studies show that when the mechanical loading effect of total body weight is accounted for, fat mass actually has a negative effect on bone health.^3^, ^4^ Recent epidemiological evidence also reveals that the relation between obesity and bone health may be site dependent.^5^ Obesity has been shown to increase the risk of fractures at the ankle and upper leg in postmenopausal women,^5^ but how it affects the risk in the vertebral column is still not clear.

Obesity is often believed to be beneficial to bone health because of the positive effect of mechanical loading conferred by body weight on bone formation.^6^ However, adipose tissue may have negative effects on bone metabolism.^6^ A number of previous studies have shown that fat mass was associated with a decrease of bone mass and bone quality at spine,^7^, ^8^, ^9^ leading to lower vertebral bone strength. The interaction of the different effects of mechanical loading and adiposity is still unclear.^10^ The underlying mechanism between obesity and bone health is likely to be complex, and may be different in men and women. Obesity in men is more characterized by central adiposity in comparison with women. Visceral adipose tissue (VAT) is particularly detrimental to bone health as it is associated with a number of hormones and cytokines that contribute to bone loss.^6^ A number of studies have shown that obesity is more consistently associated with increased prevalence of vertebral fracture when obesity is assessed using visceral fat mass.^11^, ^12^, ^13^ On the other hand, obesity has been found to be associated with increases in vertebral fracture risk in women, but not in men.^11^, ^13^, ^14^, ^15^, ^16^ Waist circumference (WC) has been a reliable clinical parameter for predicting visceral fat,^17^ whereas BMI has a stronger correlation with nonabdominal and abdominal subcutaneous fat.^17^ The correlation between obesity and the risk of vertebral fracture is likely to be dependent on whether obesity is measured by BMI or WC. There is thus a need to clarify such correlation.

Vertebral body strength is related to its BMD and the geometry of the bone.^18^, ^19^, ^20^, ^21^ Previous studies have examined how fat mass influences vertebral body BMD, which is only a “proxy measure” of the risk of fractures.^13^, ^16^, ^18^ It would also be useful to examine how fat mass affects the geometry of the vertebrae. The smaller vertebral size in women has been suggested as one of the reasons for the higher prevalence of vertebral fractures in women.^22^ However, there is no information about how obesity may affect vertebral body geometry. There is clearly a need to study such a relationship, as it would provide additional insights into how fat mass may affect bone strength and potentially the risk of vertebral fractures.

Although the risk of vertebral fracture is possibly related to mechanical loading and adiposity as discussed above, various other clinical factors will need to be considered to provide an accurate prediction of the risk of vertebral fractures. They may include history of prior fractures, age, gender, smoking, alcohol use, glucocorticoid use, rheumatoid arthritis, and secondary osteoporosis. The fracture risk assessment tool (FRAX) has been developed to evaluate osteoporotic fracture risk in untreated postmenopausal women and men aged >50 years,^23^ although the algorithm is not specifically developed for vertebral fractures. Some previous studies attempted to predict vertebral fracture risk.^24^, ^25^ They showed that fracture risks are related to morphological factors such as vertebral size or kyphosis. But clinically this information may not be available for fracture prediction. Their sample sizes were also generally small with limited power. This study will further explore the prediction of vertebral fractures considering a range of clinical factors as used in FRAX.

The aim of this study was (i) to examine the association between obesity and the risk of vertebral fracture, and whether this association was influenced by the methods of measuring obesity, (ii) to predict the risks of vertebral fractures using various clinical factors, and (iii) to study how vertebral BMD and geometry, which are both related to vertebral strength, are associated with body fat measures.

## Participants and Methods

### Study design and sample

The UK Biobank is a health resource aiming to provide data for researchers around world to study the cause of a wide range of diseases such as cancer, cardiovascular diseases, diabetes, arthritis, osteoporosis, eye disorders, depression, and dementia (https://www.ukbiobank.ac.uk). The UK Biobank is based on a prospective cohort consisting of around 500,000 UK volunteer participants aged 40 to 69 years who were first recruited and assessed from 2006 to 2010. Subsets of this original cohort were then repeatedly assessed over several time periods. The current study was based on data sets collected from two time periods: 2006 to 2010 and 2014 to 2019. It was conducted in November 2016 after approval was obtained to access the data.

The full data set consisted of 502,543 participants (229,138 men and 273,405 women) aged 40 to 69 years who were assessed using a self‐completion questionnaire and physical measurements from 2006 to 2010. The current study used this data set to examine the incidence of vertebral fractures in participants with different body weights. The data subset was a subset of this cohort, consisting of 5189 participants (2473 men and 2716 women) who were followed up in an imaging study (from 2014 to 2019) that provided DXA data of the body. This allowed us to further study BMD and the geometry of the vertebrae of the participants, as these data were not available for every participant in the full data set.

### Clinical information from the full data set

#### Anthropometric measurements

Height (standing), weight, and WC were obtained for all participants.

#### Incidence of fractures

Each participant was asked to fill in a self‐completion questionnaire in baseline assessment which included questions asking whether they had fractured/broken bones in the last 5 years and where the fractured bone sites were (eg, spine, hip, wrist, leg, ankle, arm, or others).

#### Other information

Categorical data, including smoking status (never, previous, or current smoker), daily alcohol consumption of three or more units (yes or no), history of rheumatoid arthritis (yes or no), secondary osteoporosis (yes or no), type 2 diabetes (yes or no), hormone‐replacement therapy (yes or no), and menopause (yes or no) were obtained from a self‐completion questionnaire.

### Imaging information from the data subset

#### Vertebral body BMD and geometry

DXA images (GE‐Lunar iDXA, Madison, WI, USA) were collected to obtain numerical measures of vertebral body size and areal BMD at the whole spine (C4 to L4) and the lumbar spine (L1 to L4) in the anteroposterior (AP) direction. The measures from the lumbar spine AP scan included L1 to L4 BMD, the L1 to L4 area (ie, the estimated projected area of L1 to L4 in the AP scan), L1 to L4 average height (ie, the vertebral height from the bottom of L4 to the top of L1), and L1 to L4 average width (ie, the average width of the four lumbar vertebrae L1 to L4). The measures from the whole‐spine AP scan included the spine BMD and the spine bone area. The vertebral body BMD and geometry data were obtained from 5189 participants (men = 2473, women = 2716).

#### Body composition

Body composition data were also obtained from this data subset. The measures used in this study included trunk fat mass, VAT mass, and limb fat mass, which is the sum of leg fat mass and arm fat mass. These measurements were not normalized to body weight or height.

### Data analysis

Participants (*N* = 502,543) were categorized into underweight, normal weight, and obese using BMI and WC. When BMI was used, both male and female participants were categorized according to the same criteria: underweight (BMI < 25 kg/m^2^), normal weight (25 kg/m^2^ ≤ BMI < 30 kg/m^2^), and obese (BMI ≥30 kg/m^2^). When WC was used, male and female participants were categorized using different criteria. Women were categorized as underweight (WC <80 cm), normal weight (80 cm ≤ WC < 88 cm), and obese (WC ≥88 cm); men were categorized as underweight (WC <94 cm), normal weight (94 cm ≤ WC < 102 cm), and obese (WC ≥102 cm).^26^

The association of the various categories of BMI and WC with incidence of vertebral fracture was examined in men and women using chi‐square tests.

The relationship between vertebral fractures and various clinical risk factors was studied using full data sets, including age, gender, body weight, height, history of hip and other limb fractures (they were studied separately as the risks of fractures were site‐dependent^14^), smoking, alcohol consumption, rheumatoid arthritis, type 2 diabetes, and secondary osteoporosis. The significance of these relations was examined using chi‐square tests for category data and logistic regression for continuous data. The ORs of each risk factor were determined.

Multivariate logistic regression was employed to predict the risks of vertebral fractures using the clinical factors identified above (enter method). However, only those statistically significant clinical factors were entered into the regression equation.

The imaging data subset provided information that allowed us to study fat mass, vertebral body BMD, and geometry, which was not available in the full data set. Linear regressions were employed to look at how BMD and geometry were related to trunk fat mass, VAT, and limb fat mass. Each of these fat mass measures was entered into regression analysis individually, while using age, weight, height, smoking status, hormone‐replacement therapy (for women only), and menopause (for women only) as covariates. Linear regression analysis was conducted on men and women separately. Multicollinearity between independent variables was checked by a variance inflation test (VIF <10).

SPSS 22.0 (IBM, Armonk, NY, USA) was used for all statistical analyses. Data from any participant with missing values were not included in the statistical analyses. The level of statistical significance was set at *p* < 0.05.

## Results

### Obesity and risk of vertebral fracture

Characteristics of the participants in the full data set and the imaging subset are provided in Tables 1 and 2, respectively. The ethnic background for the majority of participants was white (94.1% for baseline assessment and 96.9% for imaging study).

**Table 1:** Characteristics of Participants in the Full Data Set (Mean ± SD)

	Men (*N* = 229,138)	Women (*N* = 273,405)
Age	56.75 ± 8.19	56.35 ± 8.00
Weight (kg)	85.93 ± 14.37	71.46 ± 14.09
Height (m)	1.76 ± 0.68	1.62 ± 0.63
BMI (kg/m^2^)	27.84 ± 4.25	27.09 ± 5.19
Waist circumference (cm)	96.96 ± 11.35	84.72 ± 12.55
Previous smoker	87,614	85,458
Current smoker	28,612	24,367
Rheumatoid arthritis (yes)	1706	3952
Secondary osteoporosis (yes)	3041	6205
Type 2 diabetes (yes)	2030	1347
Menopause (yes)		165,411
Hormone‐replacement therapy (yes)		103,921

**Table 2:** Characteristics of Participants in the Data Subset (Mean ± SD)

	Male (*N* = 2473)	Female (*N* = 2716)
Age	61.89 ± 7.09	60.82 ± 7.17
Weight (kg)	84.51 ± 13.39	69.50 ± 12.74
Height (m)	1.76 ± 0.66	1.63 ± 0.63
BMI (kg/m^2^)	27.28 ± 3.99	26.38 ± 4.86
Waist circumference (cm)	93.85 ± 10.23	82.29 ± 11.59
Visceral adipose tissue mass (g)	1698.41 ± 949.57	780.51 ± 583.32
Trunk fat mass (g)	15,378.56 ± 6199.79	14,005.97 ± 6026.61
Limb fat mass (g)	8684.01 ± 3056.76	12,024.89 ± 3998.66
L1 to L4 BMD (g/cm^2^)	1.25 ± 0.19	1.14 ± 0.18
L1 to L4 area (cm^2^)	66.06 ± 6.23	54.19 ± 5.20
L1 to L4 average width (cm)	4.64 ± 0.39	4.08 ± 0.72
L1 to L4 average height (cm)	14.24 ± 0.82	13.30 ± 0.82
Spine BMD (g/cm^2^)	1.19 ± 0.15	1.02 ± 0.15
Spine bone area (cm^2^)	212.03 ± 25.47	182.27 ± 20.48
Current and previous smoker	1072	967
Menopause (yes)		2014
Hormone‐replacement therapy (yes)		1073

There were 479 vertebral fractures in 229,138 male participants and 645 vertebral fractures in 273,405 female participants in the previous 5 years, which resulted in an incidence rate of vertebral fracture at 4.2 per 10,000/ year in men and 4.7 per 10,000/ year in women.

Chi‐square analysis was conducted on BMI data from 496,812 (226,945 men and 269,867 women) participants out of 502,543 participants and WC data from 500,383 (228,062 men and 272,321 women) participants out of 502,543 participants because of missing data. There was no significant association between BMI and incident vertebral fracture in men (χ^2^ = 0.94, *p* = 0.625) or in women (χ^2^ = 4.28, *p* = 0.118; Table 3). There was a significant association between WC and incident vertebral fracture in men (χ^2^ = 8.51, *p* = 0.014), but not in women (χ^2^ = 0.71, *p* = 0.701; Table 4). Obese men (WC ≥102 cm) had higher vertebral fracture incidence (5.0 per 10,000/year) than normal weight men (3.7 per 10,000/ year) and underweight men (3.8 per 10,000/ year).

**Table 3:** Contingency Table Showing the Number of Vertebral Fractures in Different BMI Categories in Men and Women

	BMI (kg/m^2^)			
	<25	25 to 30	≥30	Total
Male				
Vertebral fracture	119	223	128	470
No vertebral fracture	56,634	112,023	57,818	226,475
Total	56,753	112,246	57,946	226,945
Female				
Vertebral fracture	267	227	131	625
No vertebral fracture	105,405	99,656	64,181	269,242
Total	105,672	99,883	64,312	269,867

χ^2^ = 0.94, *p* = 0.625 for male; χ^2^ = 4.28, *p* = 0.118 for female.

**Table 4:** Contingency Table Showing the Number of Vertebral Fractures in Different Waist Circumference Categories in Men and Women

	Waist circumference (cm)			
Men	<94	94 to 102	≥102	Total
Vertebral fracture	176	124	174	474
No vertebral fracture	91,851	66,243	69,519	227,588
Total	92,016	66,361	69,685	228,062
Women	<80	80 to 88	≥88	Total
Vertebral fracture	237	168	237	642
No vertebral fracture	104,406	68,168	99,105	271,679
Total	104,643	68,336	99,342	272,321

χ^2^ = 8.51, *p* = 0.014 for men; χ^2^ = 0.71, *p* = 0.701 for women.

The ORs of the various clinical risk factors are given in Table 5. All these factors were entered into the logistic regression equation, with the exception of alcohol consumption and type 2 diabetes, which were not shown to be significantly related to vertebral fracture risks. The logistic regression model was found to be statistically significant (omnibus test, *p* = 0.000), and was therefore a good predictor of vertebral fractures.

**Table 5:** Coefficients (*B*) of the Various Predictive Variables in the Logistic Regression Equation and Odds Ratios of These Variables

Predictive factor	*B* (SE)	OR [95% CI]
Constant	−3.690 (0.836)	
Age (years)	0.011 (0.004)**	1.012 [1.004 to 1.019]
Gender^a^	0.221 (0.087)*	1.248 [1.052 to 1.479]
Weight (kg)	−0.005 (0.002)*	0.995 [0.991 to 1.000]
Height (cm)	−0.019 (0.005)**	0.981 [0.971 to 0.990]
History of hip fracture^b^	2.419 (0.184)**	11.237 [7.833 to 16.121]
History of fractures other than hip and vertebrae^b^	1.428 (0.066)**	4.169 [3.662 to 4.747]
History of smoking^b^	0.275 (0.061)**	1.316 [1.167 to 1.484]
Rheumatoid arthritis^b^	0.778 (0.184)**	2.178 [1.518 to 3.126]
Secondary osteoporosis^b^	0.347 (0.183)*	1.415 [0.988 to 2.027]

*R* ^2^ = 0.001 (Cox & Snell); 0.040 (Nagelkerke). Model χ^2^(7) = 615.850; *p* = 0.000.

Significance of the predictive variables: *p < 0.05; **p < 0.01.

a Variable code (0 = female, 1 = male).

b Variable code (0 = no, 1 = yes).

### Vertebral body BMD and geometry

Because of missing values, the multiple linear regression analysis was conducted on data from 4849 participants (2277 men and 2572 women).

Vertebral body BMD and geometry generally showed negative association with VAT mass, trunk fat mass, and limb fat mass in both men and women (*p* < 0.05; Table 6). However, spine bone area appeared to show positive association with VAT mass and trunk fat mass, but its association with limb fat mass remained negative (*p* < 0.01).

**Table 6:** Association Between Fat Mass, Vertebral BMD, and Geometry in Male (*N* = 2277) and Female (*N* = 2572) Participants

	VAT mass (g)	Trunk fat mass (g)	Limb fat mass (g)
Male			
L1 to L4 BMD (g/cm^2^)	−0.036	−0.148**	−0.284**
L1 to L4 area (cm^2^)	−0.123**	−0.148**	−0.287**
L1 to L4 average width (cm)	−0.237**	−0.489**	−0.281**
L1 to L4 average height (cm)	0.085**	0.063	−0.058
Spine BMD (g/cm^2^)	−0.084*	−0.226**	−0.370**
Spine bone area (cm^2^)	0.248**	0.235**	−0.212**
Female			
L1 to L4 BMD (g/cm^2^)	0.035	−0.071	−0.450**
L1 to L4 area (cm^2^)	0.023	−0.133**	−0.420**
L1 to L4 average width (cm)	−0.018	−0.178**	−0.189**
L1 to L4 average height (cm)	0.093**	0.195**	−0.167**
Spine BMD (g/cm^2^)	0.054*	−0.086	−0.436**
Spine bone area (cm^2^)	0.346**	0.518**	−0.406**

Values are the standardized regression coefficients from linear regression models adjusted for age, weight, height, smoking status, hormone‐replacement therapy (for women only), and menopause (for women only). *p < 0.05; **p < 0.01. VAT = visceral adipose tissue.

The association of limb fat mass with vertebral body BMD and geometry, compared with VAT mass and trunk fat mass, appear to be stronger with larger correlation coefficients. It should also be noted the associations between L1‐to‐L4 BMD and VAT mass were weak and not statistically significant in both men and women (*p* > 0.05).

## Discussion

A strength of the present study is that it utilized data from a large cohort and attempted to answer the important clinical question of how obesity may affect the risk of vertebral fractures. BMI and WC are commonly used clinical measures to assess obesity, but only WC appears to influence the risk of vertebral fractures in men. Obese men with WC over 102 cm had a significantly higher vertebral fracture incidence compared with normal weight and underweight men. We also showed that trunk fat mass, VAT mass, and limb fat mass were negatively associated with vertebral body BMD and geometry, but the negative association was strongest for limb fat mass.

The current study provides important clinical information about how various clinical risk factors are related to and may predict the risk of vertebral fractures. These risk factors are in agreement with previous findings.^27^ The binary regression equation derived in the present study may be used by clinicians to predict the risk of vertebral fractures, providing information additional to FRAX which assesses the general risk of fractures. It is noteworthy to mention that a previous history of hip fractures is the most significant predictive factor among all the variables in the equation. This finding is in agreement with those of previous studies that the risks of fractures of these two body regions are closely related.^28^

The current study provides support for previous findings that obesity measured by BMI is not associated with vertebral fracture risk.^5^, ^29^ However, in previous studies, there were inconsistent observations about the effect of BMI on the risk of fractures. Some studies reported BMI was associated with increased risk,^9^, ^15^, ^16^ whereas others found BMI was negatively correlated with the risk.^14^ When obesity was measured by different measures, especially those related to central adiposity such as WC, trunk fat mass, and VAT mass, previous studies found that obesity was associated with an increased prevalence of vertebral fracture.^11^, ^12^, ^13^ This is in line with our findings from this study. Therefore, our study, together with others, suggests that central adiposity may be an important risk factor for the risk of vertebral fracture. In addition, the binary regression equation revealed that the risk of vertebral fractures is higher in men than in women, and this in general agreement with the observation reported previously.^13^ However, previous studies reported that obesity only affects the risks in women, but not in men.^11^, ^12^, ^14^, ^16^ This is in contrast to our finding that WC affects men only. The effect of obesity in different genders is likely to be affected by how we measure or define obesity. Another explanation is that we looked at the risk of vertebral fractures, whereas the previous studies examined other anatomical sites.

Our findings are also in line with a previous study that found that lumbar spine BMD was negatively associated with trunk fat mass and limb fat mass, but not with abdominal fat mass.^30^ However, some previous studies found that lumbar spine BMD was negatively correlated with VAT mass.^4^, ^7^, ^8^, ^31^ The different findings may be because of the different methods used in measuring vertebral body BMD and VAT mass. Although the data employed in the current study were based on DXA measurement,^30^ CT was used in those studies where different results were found.^4^, ^7^, ^8^, ^31^ Although DXA is a valid method to estimate body composition, it may not be as accurate as CT when assessing abdominal fat.^32^

There were few studies that examined the effect of fat mass on vertebral geometry, and their findings were inconsistent. One recent study found that whole‐body fat mass was negatively associated with AP vertebral diameter of lumbar spine in both men and women aged 60 to 64 years,^33^ whereas another study found that there was no association between total body fat mass and cross‐sectional area of lumbar vertebrae in teenagers and young adults.^34^ Our current study provided clear evidence that fat mass had a negative association with vertebral geometry in the lumbar spine and the whole spine.

The results from the imaging data subset showed that limb fat and trunk fat mass had a greater effect on vertebral body BMD and geometry than VAT fat mass, suggesting that visceral fat may have less influence on vertebral strength in comparison with other fat tissues. However, the results from the full data set showed that WC, which is related to visceral fat, is the only measure that is related to the risk of vertebral fracture in men. These two observations may appear to be in disagreement, but this clearly shows that BMD and the risk of fractures are not directly related to each other. Obese subjects have been found to have increased prevalence of vertebral fracture based on poor bone quality, despite normal BMD.^9^ The risk of fractures is clearly not affected by BMD only, but also a range of clinical factors including smoking, alcohol use, glucocorticoid use, rheumatoid arthritis, and secondary osteoporosis.^23^ Moreover, in obese patients, the accuracy of BMD measurement using DXA images has been shown to be adversely influenced by the thickness of VAT.^35^ In summary, it is suggested that BMD does not adequately assess the risk of vertebral fracture, especially in obese subjects.

In this study, we observed weak associations between fat mass and vertebral body BMD and geometry in both men and women. This implies there are other factors that may also influence BMD and geometry. Biomechanical factors may play a role in the associations between obesity and vertebral fracture risk. Spinal loads depend on trunk mass and the distance between trunk center‐of‐mass to the vertebrae, both of which was found to be significantly larger in the obese subjects.^36^, ^37^ It has been shown that for the same body weight a larger WC, which is related to increased visceral fat mass, can significantly move the center‐of‐mass forward and increase the spinal loads.^38^ It is possible that the increased spinal loads, together with the reduced BMD and smaller vertebral geometry associated with obesity, are responsible for the increased incidence of vertebral fractures.

The current study has some limitations. The incidence of vertebral fracture was obtained from self‐report questionnaires, and there was no information about how the reported vertebral fractures were diagnosed. It is possible that not all vertebral fractures were reported in the questionnaires as vertebral fracture is generally underdiagnosed.^39^ However, the incidence rate of vertebral fracture observed in the current study is comparable to a previous study that was based on medical records.^40^ This previous study found that for a UK population of 5 million adults the incidence rate of vertebral fracture was 3.2 per 10,000/ year for men and 5.6 per 10,000/ year for women, whereas our study found that the incidence rate was 4.0 per 10,000/year for men and 4.7 per 10,000/ year for women. Another limitation of the current study is that the logistic regression equation was derived from data obtained within a short period (between 2006 and 2010), and therefore does not represent the prospective risks as compared with FRAX, which provides a 10‐year risk prediction. However, the model is the only one at the moment that can assess vertebral fracture risk, and may be used clinically in conjunction with FRAX. Finally, low serum vitamin D level in the obese may be an important factor that may contribute to bone fragility,^7^ but we were unable to include this as a risk factor in our analysis because this information was not available from the UK Biobank.

## Conclusion

The results of the present study showed that obese men with WC over 102 cm had a significantly higher vertebral fracture incidence compared with normal weight (94 cm ≤ WC < 102 cm) and underweight (WC < 94 cm) men. Trunk fat mass, VAT mass, and limb fat mass were negatively associated with vertebral body BMD and geometry in men and women. BMD and geometry are related to vertebral strength, but they may not be directly related to the risk of fractures that are also influenced by other factors. The binary logistic regression equation established in this study may be clinically useful for the prediction of fracture risks.

## Disclosures

The authors have no conflicts of interest and nothing to disclose.
